# Assessment of the consequences of caregiving in psychosis: a psychometric comparison of the Zarit Burden Interview (ZBI) and the Involvement Evaluation Questionnaire (IEQ)

**DOI:** 10.1186/s12955-017-0626-8

**Published:** 2017-04-05

**Authors:** Manuel Gonçalves-Pereira, Eduardo González-Fraile, Borja Santos-Zorrozúa, Manuel Martín-Carrasco, Paola Fernández-Catalina, Ana I. Domínguez-Panchón, Paula Muñoz-Hermoso, Javier Ballesteros

**Affiliations:** 1 CEDOC, NOVA Medical School/Faculdade de Ciências Médicas, Universidade Nova de Lisboa, Lisboa, Portugal; 2 Clínica Psiquiátrica de S. José (Sisters Hospitallers), Lisboa, Portugal; 3 Institute of Psychiatric Research (Sisters Hospitallers), Bilbao, Spain; 4 Universidad Internacional de La Rioja, Logroño, Spain; 5 University of the Basque Country, UPV/EHU, Leioa, Spain; 6 Padre Menni Psychiatric Clinic (Sisters Hospitallers), Pamplona, Spain; 7 CIBER Mental Health, Vitoria, Spain; 8 Psychosocial Rehabilitation Resource (Sisters Hospitallers), Madrid, Spain; 9 Aita-Menni Hospital (Sisters Hospitallers), Arrasate-Mondragón, Spain; 10 CIBER Mental Health, Leioa, Spain

**Keywords:** Psychometrics, Caregivers, Schizophrenia, Community, Cost of illness

## Abstract

**Background:**

The Zarit Burden Interview (ZBI) was originally developed to assess the level of subjective burden in caregivers of people with dementia. The Involvement Evaluation Questionnaire (IEQ) is amongst the leading scales to assess caregiving consequences in severe mental illness. We aimed to compare the psychometric properties of the ZBI, a generic tool, and of the IEQ, a more specific tool to assess the consequences of caregiving in schizophrenia and related disorders.

**Methods:**

Secondary analyses of a 16-week, randomized controlled trial of a psychoeducational intervention in 223 primary caregivers of patients with schizophrenia or schizoaffective disorder. Psychometric properties (internal consistency, convergent and discriminative validity, and sensitivity to change) were evaluated for both ZBI and IEQ.

**Results:**

Internal consistency was good and similar for both scales (ZBI: 0.91, 95% CI: 0.89, 0.94; IEQ: 0.86, 95% CI: 0.83, 0.89). Convergent validity was relevant for similar domains (e.g. ZBI total score *vs* IEQ-tension *r* = 0.69, 95% CI: 0.61, 0.75) and at least moderate for the rest of domains (ZBI total score, personal strain and role strain *vs* IEQ-urging and supervision). Discriminative validity against psychological distress and depressive symptoms was good (Area Under the Curve [AUC]: 0.77, 95% CI: 0.71, 0.83; and 0.69, 95% CI: 0.63, 0.78 – for ZBI against GHQ-28 and CES-D respectively; and AUC: 0.72, 95% CI: 0.65, 0.78; and 0.69, 95% CI: 0.62, 0.77 – for IEQ against GHQ-28 and CES-D respectively). AUCs against the reference criteria did not differ significantly between the two scales. After the intervention, both scales showed a significant decrease at endpoint (*p*-values < 0.001) with similar standardised effect sizes for change (-0.36, 95% CI: -0.58, -0.15 – for ZBI; -0.39, 95% CI: -0.60, -0.18 – for IEQ).

**Conclusions:**

Both ZBI and IEQ have shown satisfactory psychometric properties to assess caregiver burden in this sample. We provided further evidence on the performance of the ZBI as a general measure of subjective burden.

**Trial registration:**

(ISRCTN32545295).

## Background

Schizophrenia is a common and often devastating psychiatric disorder that affects around 0.3–0.7% of people at some point in their life [1]. A recent World Health Organization (WHO) report estimates about 21 millions of people affected by the disease worldwide [2]. Schizophrenia has a major impact on the patient, namely on their ability to carry on day-to-day activities, on the informal caregiver and the wider society [3].

Families constitute a main support for the patient, often providing economic maintenance, health assistance, and supervision of daily tasks [4]. Family caregivers of adults with schizophrenia spend an average of 6–9 h per day providing care and attention [5]. Even if caregiving can be a positive experience [6], it is most often associated with negative consequences, including physical and mental overload, which may be related to psychological morbidity (e.g. anxiety, depression) [7]. Negative consequences were described as “caregiver burden” more than half a century ago [8]. Given their impact on both caregivers and patients, the interest in understanding and managing caregiver burden has grown exponentially, not only in schizophrenia [9] but also in all other chronic health conditions [10].

Caregiver burden includes objective and subjective components [11]. Objective burden mostly concerns the tangible tasks caregivers usually perform to help the patient. Subjective burden mainly regards personal appraisals of objective burden, including emotional reactions to the caregiving experience [12].

The Zarit Burden Interview (ZBI) and the Involvement Evaluation Questionnaire (IEQ) are amongst the most reputable instruments to assess caregiving consequences, although their characteristics differ.

The ZBI was originally developed to assess the level of subjective burden in caregivers of people with dementia [13]. It has been extensively validated in caregivers of frail old people with neuropsychiatric disorders [14], having become a gold-standard in this field. Being arguably the most widely used measure of caregiver burden [15], it has also been used in schizophrenia research to describe the experiences of caregivers [16, 17] and to assess the efficacy of family psychosocial interventions [18–21]. However, most research with the ZBI in schizophrenia has been conducted in the Hispanic or non-Western world [17–26] and the validity and reliability of the ZBI in schizophrenia remains to be established [15, 27]. This led to the recent issue of the Schizophrenia Caregiver Questionnaire (SCQ), adapting the ZBI to this specific clinical context [15] and in different cultures [28, 29]. Overall, given its widespread - albeit sometimes unspecific - use across health conditions, the original ZBI has been considered a generic measure of burden [29–31].

The IEQ is amongst the leading scales to assess caregiving consequences in severe mental illness [32]. It was cross-culturally validated in Europe in the EPSILON study [33, 34], used in longitudinal observational research [35] and in very different cultures [35–40]. While also validated in affective [41] or eating disorders [42], it is mainly considered one of the standards for the evaluation of caregiving consequences in schizophrenia, together with the Family Problems Questionnaire [43] or the Experience of Caregiving Inventory [6]. Despite some contributions [44–46], sensitivity to change has been less explored.

To our knowledge, concurrent use of the original ZBI (as a generic burden measure) and IEQ (as a specific assessment of caregiving consequences and burden in severe mental illness, including schizophrenia) has never been reported in caregivers of psychotic patients.

In this study we aimed to evaluate and compare psychometric properties of the ZBI and the IEQ (reliability, validity, and sensitivity to change) in a large sample of caregivers of psychotic patients from two Southern European countries. By doing so, we were interested in further testing the usefulness of the ZBI as a generic burden measure, applicable to a range of clinical situations.

## Methods

### Research design and study population

This is a secondary analysis of data from the multicentre EDUCA-III study, a 16-week randomised controlled trial that assessed the efficacy of a group psychoeducational intervention on caregiver burden in schizophrenia (trial registration: ISRCTN32545295) [47].

The intervention consisted of a cognitive-behavioural program designed to improve primary caregivers’ education and skills regarding psychosis, aiming to promote stress management overall. The program was administered over 12 weekly group sessions, lasting 90–120 min each, as detailed elsewhere [47]. Throughout these sessions, caregivers received information about schizophrenia (clinical issues, treatment) and were trained in e.g., caregiving and communication skills, how to address challenging behaviours, the ability to look for and enjoy pleasant events, to seek social support, to use relaxation techniques. The program required active participation (e.g. role playing) and focused on problem-solving, helping caregivers to challenge negative beliefs through positive reframing, and to develop more adaptive caregiving styles. The intervention materials are freely available in Spanish (http://www.fundacion-iip.org/IIP/lineas-investigacion/sobrecarga-cuidador-educa-III.html).

Primary caregivers were eligible if they were (a) 18 years or older, (b) currently caring for a relative with a diagnosis of schizophrenia or schizoaffective disorder, (c) not paid and spending a minimum of 4 h/week caring for the patient. The patients (care receivers) should have been diagnosed at least 2 years before trial recruitment and receiving appropriate outpatient clinical care. Caregivers without time to attend the intervention, or currently receiving or having recently received any standardised psychoeducational intervention were excluded. A total of 223 caregivers were randomised to intervention or control condition, and assessed at baseline and at endpoint (~4 months since baseline). Regarding the present study, these 223 caregivers were all included in the analysable sample.

### Rating scales

In order to meet the aims of this secondary analysis, we used the two main outcome measures of the EDUCA-III trial, i.e. those assessing caregiver consequences (ZBI and IEQ). To document their discriminative validity, two other EDUCA-III measures were also used as reference criteria to assess psychological distress (the General Health Questionnaire, 28 item version; GHQ-28) and depressive symptoms (the Center for Epidemiologic Studies Depression Scale; CES-D).

#### Zarit Burden Interview (ZBI)

The ZBI includes 22 items recorded in a 0–4 Likert scale (total score range 0 to 88). They refer to problems arising in several domains: health and well-being, personal and social life and finances. As the ZBI assesses the feelings/thoughts of informal caregivers on the impact of the disease on their lives, it is considered to focus on the subjective component of burden of care. Higher scores on the ZBI mean higher burden [13]. Besides its total score, which is used most often, personal strain (12 items) and role strain (6 items) domains have been proposed in dementia [48]. We used translations of the ZBI validated in Spain [49] and Portugal [50] in caregivers of people with dementia. In the present study, and although it is not a standard instruction, respondents were asked to focus on the previous 4 weeks to answer the items.

#### Involvement Evaluation Questionnaire (IEQ)

The IEQ includes a 31-item core module questionnaire recorded in a 0–4 Likert scale, assessing the frequency of a broad array of consequences of caregiving (e.g. feelings, cognitions, behaviours) within the previous 4 weeks. Besides its total score, the IEQ includes four subscales: worrying (6 items), urging (8 items), tension (9 items) and supervision (6 items). *Tension* refers to the strained interpersonal atmosphere between patient and relatives, *supervision* to the caregiving tasks of ensuring and guarding related to e.g. patient’s intake of medication or dangerous behaviours, *worrying* to painful cognitions and concerns about patient’s safety or future, and *urging* to issues related to activating and motivating the patient. Higher scores mean higher levels of caregiver consequences [34]. The IEQ was translated and validated in Spanish [34] and Portuguese [39].

#### General Health Questionnaire 28 items (GHQ-28)

The GHQ-28 includes 28 items. In the present study, items were scored according to the classical 0011 method, with a total score range of 0–28. Higher scores on the GHQ-28 mean higher levels of psychological distress [51]. There are validated versions for Spanish [52] and Portuguese [53] populations. The GHQ-28 can also be used in epidemiological studies as a screening for minor psychiatric morbidity caseness (clinically significant anxiety and/or depression). The 4/5 and 5/6 cut-off values are often used [51] and 4/5 is the modal value according to the GHQ Manual, but higher values have been reported [54, 55]. However, evidence of cut-off validity varies across populations. In Spain, 5/6 and 6/7 were proposed [52], while no cut-off validation studies have been conducted in Portugal.

#### Center for Epidemiologic Studies Depression Scale (CES-D)

The CES-D is a 20-item questionnaire that assesses depressive feelings and related behaviours during the past week. Items are rated on a 0–3 Likert scale, giving a total range from 0 to 60. Higher scores on the CES-D mean higher levels of depressive symptoms [56]. There are Spanish [57] and Portuguese Versions of CES-D [58]. Adopting a 15/16 cut-off, the CES-D has also been used as a screening tool for depression, with good sensitivity and specificity, and high internal consistency across age ranges [59]. This approach seems less contentious than the use of specific GHQ cut-offs.

### Statistical analysis

Means and SDs describe continuous variables. Frequencies and percentages describe categorical variables.

#### Reliability

Internal consistency of the burden scales was evaluated for the whole sample of caregivers at baseline by Cronbach’s coefficient α. A value ≥ 0.80 was considered the minimum level to attain [60].

#### Convergent validity

Convergent validity between the ZBI (total and domain scores) and the IEQ (total and subscale scores) was calculated for the whole sample of caregivers at baseline with the Pearson correlation coefficient, after checking linear association.

#### Discriminative validity: receiver operator characteristic (ROC) and area under the curve (AUC) analyses

We used ROC and AUC analyses [61] in the whole sample of caregivers at baseline to assess the ability of both burden scales to discriminate among levels of psychological distress, as reported by the GHQ-28 according to a range of possible cut-off values [62], and CES-D (reference cut-off values 15/16) [56]. The ROC curve plots the sensibility and specificity of the burden scales for every possible cut-off point against the reference criterion (in our case the GHQ-28 and CES-D scales) and gives the AUC as a summary performance index. The AUC is interpreted as the probability to correctly discriminate among subjects categorized by the cut-off values of the reference scales (GHQ-28 and CES-D). A value of 0.5 for the AUC implies that discrimination is not improved beyond chance (flipping a coin) whereas a value of 1 implies a perfect discrimination. We used the following AUC values to interpret the discriminative validity: [0.50, 0.60), bad test; [0.60, 0.75), ordinary or regular test; [0.75, 0.90), good test; [0.90, 0.97), very good test; [0.97, 1.00], excellent test. AUC values for both burden scales were compared with DeLong’s test [63].

#### Sensitivity to change or responsiveness

It was assessed by the ZBI and IEQ score changes for the intervention group between baseline and endpoint scores (~ 4 months). We used the within-group standardised effect size [64] that takes into account the non-independent difference between the baseline and endpoint scores.

All analyses were performed with Stata v14 (StataCorp, College Station, TX, 2015) and/or R 3.2.3 (R Foundation for Statistical Computing, Vienna, Austria, 2015) with the library *pROC* [65].

## Results

### Sample description

The EDUCA-III trial recruited 223 caregivers (109 randomised to intervention and 114 to control) from 23 research sites under the aegis of Sisters Hospitallers, in Spain and Portugal. Caregivers were predominantly women (76%), married (62%), with a mean age of 60 years (SD = 11). They presented a mean caregiving exposure of 15 years (SD = 10), and a mean caregiving load of 5 h per week (SD = 1). The corresponding patients (care receivers) presented a diagnosis of schizophrenia (86%), or schizoaffective disorder (14%). A detailed sample description can be found elsewhere [47].

### Reliability

The internal consistency (Cronbach’s alpha) for the ZBI (*n* = 223) was 0.91 (95% confidence interval [CI]: 0.89, 0.94), whereas for the IEQ (*n* = 223) it was 0.86 (95% CI: 0.83, 0.89). Both values were above the minimum level established (0.80).

Cronbach’s alphas for ZBI subscales were 0.84 (95% CI: 0.81, 0.87) for personal strain and 0.86 (95% CI:0.83, 0.89) for role strain. Reliability for the IEQ subscales was 0.71 (95% CI:0.66, 0.77) for tension, 0.77 (95% CI:0.73, 0.82) for supervision, 0.77 (95% CI:0.73, 0.82) for worrying and 0.72 (95% CI:0.67, 0.76) for urging.

### Convergent validity

**Fig. 1:**
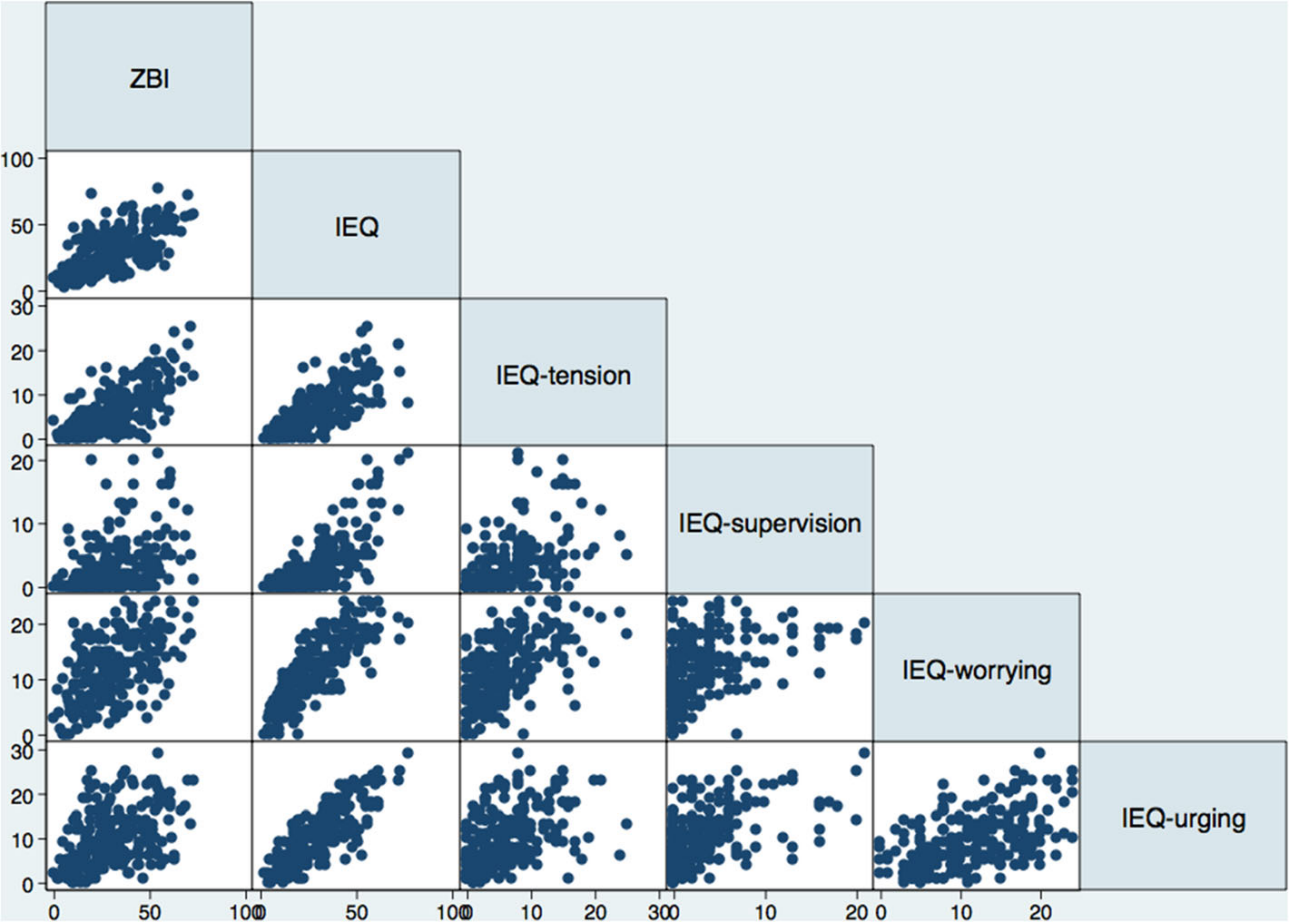
Correlations between ZBI and IEQ scales

Table 1 shows the Pearson’s correlation coefficients between ZBI total score and IEQ total and subscale scores. All correlations are linear (Fig. 1) and present at least a moderate level (*r* ≥ 0.40; all *p*-values < 0.0001). The higher correlation (*r* = 0.69) was found between the ZBI and the tension subscale of the IEQ. Regarding correlations between the ZBI domains (personal and role strain), and IEQ total and subscale scores, the higher coefficient was found between ZBI personal strain and IEQ tension (*r* = 0.66; *p* < 0.0001).

**Table 1:** Pearson’s correlations between ZBI and IEQ scales

	ZBI (*n* = 223) *r* (95% CI)	ZBI Personal Strain *r* (95% CI)	ZBI Role Strain *r* (95% CI)
IEQ	0.63 (0.54, 0.70)	0.51 (0.41, 0.60)	0.55 (0.46, 0.64)
IEQ-tension	0.69 (0.61, 0.75)	0.66 (0.57, 0.72)	0.57 (0.48, 0.65)
IEQ-supervision	0.40 (0.28, 0.51)	0.34 (0.21, 0.45)	0.32 (0.20, 0.43)
IEQ-worrying	0.55 (0.45, 0.63)	0.44 (0.32, 0.54)	0.54 (0.44, 0.62)
IEQ-urging	0.43 (0.32, 0.53)	0.29 (0.16, 0.40)	0.38 (0.27, 0.49)

*ZBI* Zarit Burden Interview, *r* Pearson’s correlation coefficient, *CI* confidence interval, *IEQ* Involvement Evaluation Questionnaire

### Discriminative validity

**Fig. 2:**
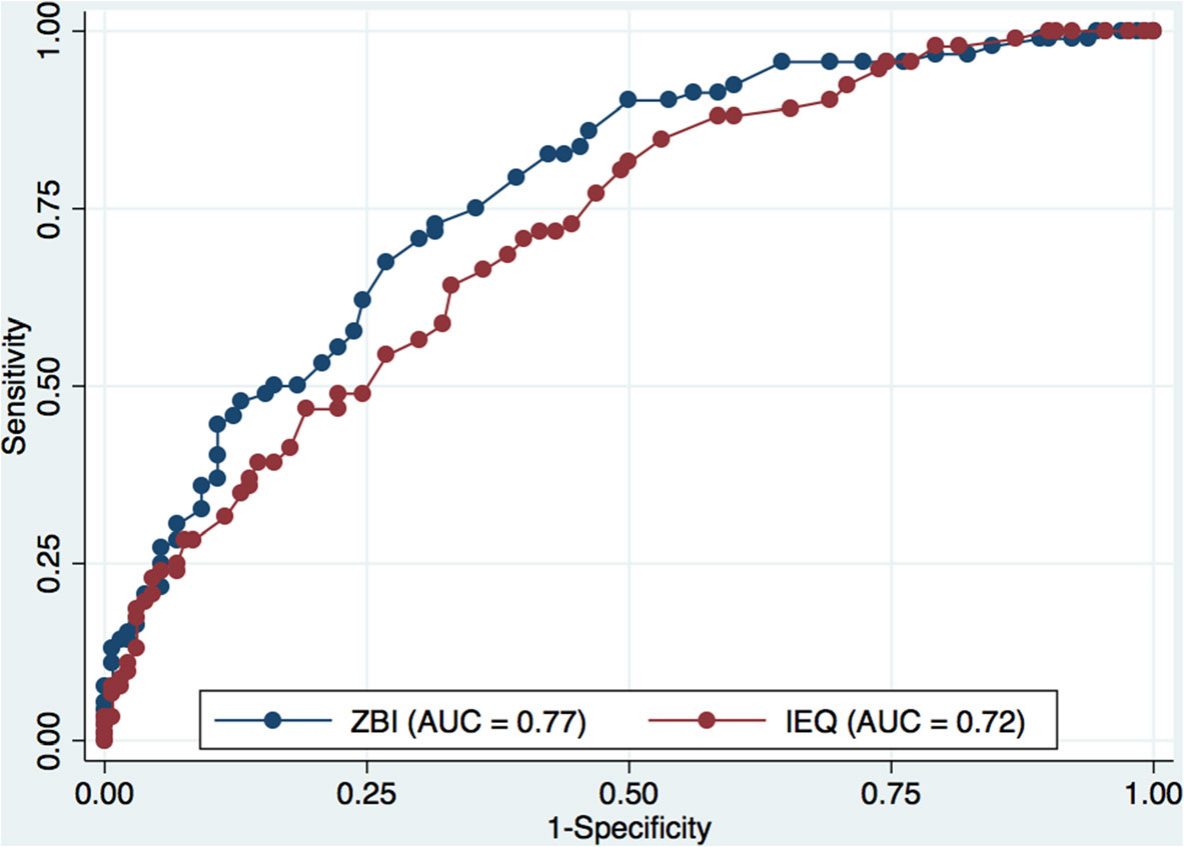
ROC and AUC analyses of ZBI and IEQ compared against the GHQ-28 at 4/5 cut-off point

Table 2 shows the ROC and AUC analyses of both ZBI and IEQ as compared to (i) a range of the GHQ-28 cut-off points (*n* = 222), and (ii) the CES-D at 15/16 cut-off point (*n* = 197). Figure 2 shows the ROC and AUC analyses of both ZBI and IEQ as compared against the GHQ-28 at the most usual 4/5 cut-off point (130 [59%] subjects presented a GHQ-28 score ≤ 4; 92 [41%] subjects presented a GHQ-28 score ≥ 5). The AUC for the ZBI was 0.77 (95% CI: 0.71, 0.83), whereas for the IEQ was 0.72 (95% CI: 0.65, 0.78). These AUCs did not differ significantly (*p*-value = 0.25).

**Table 2:** Receiver Operating Curve (ROC) analysis of the ZBI and IEQ against the GHQ-28 and CES-D

GHQ-28 cut-off points	AUC – ZBI	AUC – IEQ	*p*-value ^1^
3/4	0.773	0.725	0.298
4/5	0.771	0.718	0.253
5/6	0.745	0.699	0.345
6/7	0.744	0.711	0.491
CES-D cut-off point			
15/16	0.696	0.696	0.991

^1^ *p*-value for the comparison of AUC’s

ROC and AUC analyses (*n* = 197) of both ZBI and IEQ as compared against the CES-D at 15/16 cut-off point show that 115 (58%) subjects presented a CES-D score ≤ 15 and 82 (42%) subjects presented a CES-D score ≥ 16. The AUC for the ZBI was 0.69 (95% CI: 0.63, 0.78), whereas for the IEQ was 0.69 (95% CI: 0.62, 0.77). These AUCs did not differ significantly (*p*-value = 0.99).

Overall, both scales presented similar performance with values corresponding to a regular to good test when discriminating between levels of psychological distress (GHQ-28), or depressive symptoms (CES-D).

### Sensitivity to change

Table 3 presents the results of the sensitivity to change (~ 4 months) in subjects randomly allocated to the intervention (*n* = 109). Both scales’ scores showed a significant decrease at endpoint (*p*-values < 0.001) presenting a similar and moderate standardized effect size for change (-0.36 for the ZBI, -0.39 for the IEQ).

**Table 3:** Sensitivity to clinical change at 4 months since baseline

	ZBI (*n* = 85)	IEQ (*n* =86)
Baseline mean score (SD)	32.3 (16.0)	31.1 (16.2)
Endpoint mean score (SD)	27.7 (13.4)	26.6 (14.2)
Mean change endpoint – baseline scores (95% CI)	4.6 (1.9, 7.3)*	4.5 (2.0, 7.0)*
Within-group effect size (95% CI)	−0.36 (−0.58, −0.15)	−0.39 (−0.60, −0.18)

*ZBI* Zarit Burden Interview, *IEQ* Involvement Evaluation Questionnaire, *SD* standard deviation, *CI* confidence interval

* *P*-value < 0.001

## Discussion

### Main findings

The main aim of this study was to evaluate and compare the psychometric properties of the ZBI and the IEQ in a large sample of caregivers of psychotic patients from Spain and Portugal. The EDUCA III intervention study was a golden opportunity to conjointly assess aspects of the performance of both measures, including sensitivity to change. We found good internal consistency for both scales. Correlations between ZBI and IEQ total scores were moderate, demonstrating adequate convergent validity. As expected, only to a certain degree did their results converge, especially regarding information from IEQ domains. We found higher values on correlations between ZBI and IEQ *tension*, and ZBI and IEQ *worrying*, both being ‘interpersonal’ IEQ domains and the most related to subjective burden. Noteworthy, correlations between ZBI and IEQ total scores, and between ZBI total score and IEQ *tension* were similar. According to Schene et al. [32], perhaps the integrated evaluation of caregivers tasks and problems is best represented by the tension domain. This is corroborated by the fact that the IEQ item on global burden (which reads similar to the ZBI one) contributes both to the IEQ total score and to the tension subscale [32].

The AUC analysis showed an appropriate ability of the ZBI and the IEQ to discriminate between levels of psychological distress. Finally, both scales proved to be valid and reliable tools overall to longitudinally evaluate the impact of psychosis in this sample of caregivers.

Sensitivity to change is one of the psychometric properties more difficult to document. In schizophrenia caregiving, even a robust measure such as the IEQ is no exception to this, albeit having been conceived to ensure adequate sensitivity to change [32]. The ZBI, could be expected to perform less well in psychoses at this regard: some ZBI items relate to issues as guilt or stigma, which are less prone to change over time [32], even by means of an effective family intervention. The choice of any caregiver outcome requires thoughtful consideration of the likely effects of the intervention [14], and this was our concern during the design of the EDUCA III trial. We could not exclude, at the beginning, that the ZBI would be more sensitive to some aspects of the intervention package (e.g. therapist and peer-related emotional support), whereas the IEQ could be more sensitive to others (e.g. behavioural changes). It may also be that the specification of a 4-week time frame for the ZBI, overcoming the lack of a specific recall period, contributed to a better performance of this measure regarding sensitivity to change.

### Comparison with other studies using the ZBI in schizophrenia

While the ZBI has been used in schizophrenia caregiver related research in e.g. South American, Hispanic north-American, African, Eastern populations [17–26, 47, 66], only one study seemed to report internal consistency in the corresponding sample (Cronbach’s alpha = 0.89) [25]. Others reported some contributions to construct or factorial validity [23, 24], or evidence suggesting sensitivity to change [18–21, 26]. Overall, these studies tended to support the ZBI as a measure of subjective burden in schizophrenia, able to discriminate between levels of strain and to assess the efficacy of interventions designed to reduce caregiver burden, even lacking schizophrenia-specificity. Our results add to this line of research by further detailing some psychometric properties of the ZBI in the field of psychoses, in an unprecedented way.

### The ZBI as a generic measure of subjective burden in a range of clinical context?

There were different reasons for our interest in testing the ZBI, which was never fully validated as a specific measure in psychosis caregiving, concurrently with the IEQ, a well-validated specific tool in the area. We had experience with the ZBI in dementia, in observational [50] and intervention [47, 67] studies, and with the IEQ in psychosis, in observational [68] and intervention research [45]. Realizing that the ZBI would not be enough as primary outcome measure in a trial of persons with psychosis, we decided to use it conjointly with the IEQ. We hypothesized that ZBI and IEQ grasped different facets of caregiving in schizophrenia and related disorders. Strictly speaking, these measures are not ‘psychological tests’; they cover constructs that are diffuse, and boundaries with each other and with other constructs may be less clear [34]. On the whole, the ZBI could be seen as a general approach to a wide array of caregiving situations, and most related to subjective burden issues. The IEQ stands as a more specific approach to caregiving consequences in schizophrenia and other severe mental illnesses, also encompassing objective burden components.

Besides its tentative previous use in schizophrenia, the ZBI is being used extensively in very different clinical contexts, including studies where different populations of caregivers are compared (e.g. chronic obstructive pulmonary disease, acquired brain injury, palliative care) [69, 70]. Therefore, further documenting ZBI’s psychometric properties in schizophrenia could help to bridge existing gaps in the design of comparison studies including schizophrenia caregivers. Despite that a specific measure will always be needed to fully evaluate caregiving consequences in a given field, the ZBI may perform well as a measure to be used across some conditions, provided its psychometric properties are documented. In sum, our findings allow for an elaboration on the ZBI as a generic burden measure, partly applicable to a range of clinical situations including schizophrenia and related disorders.

### Strengths and limitations of the study

This study was a secondary analysis of the psychometric properties of two important caregiver measures (ZBI and IEQ) concurrently used in a large multicentre trial in two south-European countries. The ZBI had never been tested comprehensively in caregivers of people with psychosis neither in Spain nor Portugal. Moreover, this exploration had never been systematically conducted, as far as we are aware of, in the Anglo-Saxon world or elsewhere.

We did not replicate test-retest reliability studies for any of the two scales, as this would not be feasible in the context of the EDUCA III trial.

### Research implications

At time of writing of our study, an adaptation of the original ZBI to specifically evaluate caregiver burden in psychoses was published [15]. This revised version (the SCQ) modifies some expressions, introduces a recall period, and incorporates new items about aspects related with caregiver burden. It presents face and content validity [15], and is now beginning to be tested in multicentre studies with promising results [28]. We acknowledge this step forward regarding the evaluation of specific caregiving consequences in schizophrenia.

However, even at the light of these developments, our results remain an important contribution to this field of research. The psychometric properties of the original ZBI were overall satisfactory in our EDUCA-III trial, as compared to a schizophrenia-specific and robust measure, i.e. the IEQ. This strongly supports the original ZBI as a valid option to complement more specific instruments, whenever the research aims call for including a measure with established validity in multiple clinical contexts, facilitating comparison studies across health conditions.

## Conclusions

The ZBI and the IEQ are robust caregiver assessments, originally validated in different clinical fields (the former in dementia and frail old age populations, the latter in schizophrenia and severe mental illness in general). Both scales performed well regarding their psychometric properties (e.g. sensitivity to change) in this large sample of psychotic patients’ caregivers. As the ZBI has been increasingly used across health conditions, our study provides further evidence-base to the ZBI as a generic subjective burden measure. This may be helpful in comparison studies including caregivers of persons with psychosis

## Abbreviations

AUC: Area under the curve
CES-D: Center for epidemiologic studies depression scale
CI: Confidence interval
GHQ-28: General Health Questionnaire 28 items
IEQ: Involvement evaluation questionnaire
r: Pearson’s correlation coefficient
SCQ: Schizophrenia caregiver questionnaire
SD: Standard deviation
WHO: World Health Organization
ZBI: Zarit burden interview
